# Qualitative Characterization and Antifungal Activity of Romanian Honey and Propolis

**DOI:** 10.3390/antibiotics11111552

**Published:** 2022-11-04

**Authors:** Mihaela Laura Vică, Mirel Glevitzky, Gabriela-Alina Dumitrel, Roxana Bostan, Horea Vladi Matei, Yordanka Kartalska, Maria Popa

**Affiliations:** 1 Department of Cellular and Molecular Biology, “Iuliu Hațieganu” University of Medicine and Pharmacy, 400012 Cluj-Napoca, Romania; 2 Institute of Legal Medicine Cluj-Napoca, 400006 Cluj-Napoca, Romania; 3 Faculty of Exact Science and Engineering, “1 Decembrie 1918” University of Alba Iulia, 510009 Alba Iulia, Romania; 4 Sanitary Veterinary and Food Safety Directorate of Alba County, 510217 Alba Iulia, Romania; 5 Faculty of Industrial Chemistry and Environmental Engineering, Politehnica University of Timisoara, 300223 Timișoara, Romania; 6 Department of Microbiology and Ecological Biotechnologies, Agricultural University of Plovdiv, 4000 Plovdiv, Bulgaria

**Keywords:** bee products, physicochemical properties, antioxidant activity, zone of inhibition, fungal strains, statistical analysis

## Abstract

The purpose of this study was to review the physicochemical characterization of Romanian honey and propolis and their antifungal effect on different strains. As an indicator of environmental pollution, lead exceeded the allowed limits in two study areas. The relationship between the acidity and electrical conductivity of polyfloral honey and the antioxidant activity with the total content of phenolics and flavonoids was investigated. The antifungal activity of 13 polyfloral honey and propolis samples from North-West and Central Romania and 12 samples from Alba County was investigated against six fungal strains: *Aspergillus niger, Aspergillus flavus, Candida albicans, Penicillium chrysogenum, Rhizopus stolonifer*, *Fusarium oxysporum*. All honey and propolis samples exhibited an antifungal effect. The most sensitive strains were *P. chrysogenum* and *R. stolonifer* for honey and *P. chrysogenum* and *F. oxisporumn* for propolis. A two-way analysis of variance was used to evaluate the correlations between the diameter of the inhibition zones for the strains and the propolis extracts. Statistical analysis demonstrated that the diameter of the inhibition zone was influenced by the strain type and the geographical origin of honey and propolis. Pearson’s correlation coefficient shows a significant positive linear relationship between the diameter of the inhibition zone and the flavonoid and phenol concentration of honey and propolis, respectively.

## 1. Introduction

Honey is a sweet, viscous substance produced by bees from the nectar of flowering plants. It is produced in almost every country in the world and is a natural food, a medicine and an energy food ingredient. Honey is a natural unrefined sugar, a sweetener, which is easily absorbed by the body, providing energy. Fructose and glucose are the primary carbohydrates found in honey. Honey is essentially pure sugar containing vitamins and enzymes, without fat and with only trace amounts of protein and fiber [1]. From a nutritional point of view, in addition to a high content of sugars, there are also organic acids, amino acids, minerals, aromatic substances, etc. [2]. Being a “noble” bee product, honey is a source of therapeutic agents, having a wide range of action depending on the type, origin, composition, etc. [3,4]. The contamination of honey with heavy metals, especially lead, cadmium and copper is affected by the location of bee colonies, such as industrial areas or other areas with considerable atmospheric pollution (urban environment), the use of toxic chemicals in agriculture, dirty water, non-floral sugar sources [5], and the storage of honey in reused containers [6]. Propolis or ‘bee glue’ is a resinous substance produced by bees that mixes saliva and beeswax with exudate collected from flowers and plant buds, with which bees cover holes and cracks in the hive, defending against bacteria and other microorganisms [7,8]. There are different compounds that we can find in propolis: wax, phenolic acids, flavonoids, balsam, aromatic and essential oils. Its composition can be very different depending on the geographical area and botanical origin. Like honey, propolis is known to have antioxidant, antimicrobial, anti-inflammatory or even anti-carcinogenic effects [9,10,11].

Bee honey and propolis have been used for a long time due to the properties and health benefits of these natural bee products [12]. Today, there is a growing interest in the identification of alternative and natural antimicrobial agents. The advantage of natural products could be that they do not generate resistance, as do some synthetic antibiotics.

Honey and propolis are also known to have antioxidant capacity, in addition to their antibacterial activity. This denotes the fact that the compounds in the bee products act against free radicals [13]. The entire antimicrobial mechanism of bee products is still incompletely studied, although the antibacterial effect of bee honey is already well-known and has been studied for a long time [14,15,16]. In addition, the antifungal effect of honey from different regions has been shown in different studies, on different strains such as *Aspergillus* spp., *Alterneria* spp., *Fusarium* spp., *Microsporum* spp., *Penicillium* spp., *Rhizopus* spp. or *Candida* spp. [17,18]. Many researchers focused on the biological properties of propolis, including cytotoxic, antiherpetic, antioxidant, antimicrobial, and anti-HIV [19,20,21,22]. Its antibacterial effect has been shown in several studies [23,24,25,26,27], but the data from the literature focused less on the antifungal effect of propolis [28].

In this context, the aim of our study was to determine the physicochemical characteristics of several honey and propolis samples collected from different locations in the North-West and the center of Romania, as well as to evaluate the antifungal activity of these samples against several strains. At the same time, correlations between the physicochemical parameters and the antifungal effect of the studied samples were highlighted.

## 2. Results

### 2.1. Physicochemical Characterization of Honey and Propolis Samples

In order to investigate the quality of honey, the physicochemical parameters, presented in Table 1, Table 2, Table 3 and Table 4, have been determined.

The physical parameters are within the Romanian and European standards, with a slight increase in the value of acidity for subsamples 11 and 12 and the electrical conductivity for subsample 11.

The total phenolic and flavonoid contents in the honey samples ranged from 39.41 to 128.52 mg GAE/100 g for phenols and 1.84 to 7.39 mg QE/100 g for flavonoids. All polyfloral honey samples, collected from beekeepers, have the values of chemical parameters within the reference intervals presented in the EU [29] or Romanian national regulations [30]. The exception is the SS2 and SS4 subsamples, which have high lead content, exceeding almost twice the maximum allowed limit.

The results of the physicochemical determinations of the propolis samples are presented in Table 5 and Table 6.

In the case of propolis samples, from North-West and Central Romania and from Alba County, the identification reactions of flavones and aromatic acids were positive. We can consider the presence of these compounds in all the investigated samples together with the content of phenols and flavonoids presented in Table 5 and Table 6. The total phenolic content of the propolis samples ranged from 129.6 to 203.3 mg GAE/g, while the total flavonoid content was 61.56 to 90.54 mg QE/g.

### 2.2. Antifungal Activity of Honey and Aqueous Propolis Extracts

Table 7, Table 8, Table 9 and Table 10 show the results of the antifungal effect of the honey and propolis samples. As can be seen in Table 7 and Table 8, all honey samples showed antifungal activity against all types of strains. The diameters of the inhibition zones ranged from 7 to 12 mm. The artificial honey sample had no effect on any of the strains.

As shown in Table 7 and Table 8, the most sensitive strains to the effect of bee honey were those of *P. chrysogenum* and *R. stolonifer*, followed by strains of *Aspergillus* spp. Samples S4, S5 and S13 were the most effective regarding the antifungal effect, presenting the largest diameters of the inhibition zones.

Table 9 and Table 10 show the diameters of the inhibition zone produced by the aqueous extracts of propolis. All samples had an antifungal effect, the diameters varying between 15 and 28 mm.

As shown in Table 9 and Table 10, the diameters of the inhibition zones were larger in the case of propolis extracts than in the case of bee honey, the most sensitive fungal strains to the effect of propolis being *P. chrysogenum* and *F. oxisporum*. The aqueous extracts of propolis S4 and S5 (as in the case of honey) and S1 had the strongest antifungal effect.

The different diameters of the inhibition zones of honey and propolis can be explained by the different compositions of these products and by the different ways of inducing the antimicrobial effect. In the case of honey, the antimicrobial activity is correlated with the amount of hydrogen peroxide present as well as with the existence of additional antibacterial compounds that come from the source nectar. The antimicrobial effect of propolis can be explained by the presence of quercetin with strong antimicrobial activity, as well as by the high concentration of polyphenols and flavonoids [31,32].

### 2.3. Minimum Inhibitory Concentration (MIC)

All honey samples showed an inhibitory effect at dilutions of up to 1/16 (*w/v*) for one or more strains. The only exception was sample S11, which had no effect at the 1/16 dilution on any of the strains. Some samples showed antifungal inhibition even at a dilution of 1/32 (*w/v*), but not on all strains.

Regarding propolis samples, with the exception of samples S13 and SS6, they all had an antifungal effect up to concentrations of 6.25 mg/mL, but not for all strains. Many of the samples had an inhibitory effect at concentrations of 3.12 mg/mL on some of the strains, and samples S1, S5 and SS7 had an effect on the strains of *F. oxysporum* (sample S1) and *P. chrysogenum* (samples S5 and SS7) even at concentrations of 1.56 mg/mL.

### 2.4. Statistical Analysis

The bifactorial analysis of variance tested the simultaneous interaction of two independent variables: Romanian honey and propolis extracts and the diameter of the inhibition area for the studied strains.

For the 13 samples of honey (H) and propolis (P) collected from North-West and Central Romania, the computation of variances caused by each independent parameter, including residual dispersion caused by accidental factors, produced the following results: S_1,H_ = 6992, S_2,H_ = 4921.66, S_3,H_ = 6915.07, S_4,H_ = 6869.53, S_1,P_ = 37.636, S_2,P_ = 37.090, S_3,P_ = 37.140.77, and S_4,P_ = 36.790.21. The results of the two-way ANOVA are presented in Table 11.

As 5 degrees of freedom were found for the honey and propolis samples(ν_1_) and 12 for the strains tested (ν_2_), since F_col,H_ = 10.42 and F_col,P_ = 18.40 are both greater than F_0.05_ = 2.37; the null hypothesis that the mean values of the columns are equal was rejected. It was concluded that the honey and propolis samples from North-West and Central Romania influenced the diameter of the inhibition zone. Additionally, because F_row,H_ = 9.18 and F_row,P_ = 8.96 are also both greater than F_0.05_ = 1.92, the hypothesis that the mean values of the rows are equal was rejected and it was concluded that the type of strain affected the inhibition areas. The significance level was α = 0.05.

For the 12 samples of honey (H) and propolis (P) collected from Alba County, the ANOVA sums-of-squares: S_1,H_ = 6453, S_2,H_ = 6381.83, S_3,H_ = 6386.58, S_4,H_ = 6365.68, S_1,P_ = 35.608, S_2,P_ = 35.171, S_3,P_ = 35.098.83, and S_4,P_ = 34.848. The results of the two-way ANOVA are presented in Table 12.

As 5 degrees of freedom were found for the honey and propolis samples (ν_1_) and 11 for the strains tested (ν_2_), since F_col,H_ = 3.53 and F_col,P_ = 19.08 are both greater than F_0.05_ = 2.38, the null hypothesis was rejected. There is enough evidence to support the claim that the origin of honey and propolis samples from Alba County influenced the diameter of the inhibition zone. Furthermore, since F_row,H_ = 2.07 and F_row,P_ = 6.73 are also greater than F_0.05_ = 1.97, the hypothesis that the mean values of the rows are equal was rejected and it was concluded that the type of strain affected the inhibition areas. The significance level was α = 0.05.

In Table 13, Pearson’s correlation coefficient values show the strength of a linear association between the diameter of the inhibition zone and the flavonoid and phenol concentration of honey and propolis, respectively, for all the microorganisms studied.

The correlations between the diameter of the inhibition zone and flavonoids were low and medium, indicating, in general, a positive linear relationship between the two variables. The strength of association is smaller in the case of honey samples, compared to propolis extracts, which presents an even stronger positive correlation. The correlations between the diameter of the inhibition area and the phenols were similar, suggesting a moderate and strong positive linear relationship.

## 3. Discussion

Due to the fluctuation in the physicochemical parameters and the composition found in bee products from all over the world, each sample should be analyzed and classified according to its chemical profile. Due to the diversity of flora used by bees to produce honey and propolis, specific to each geographical area, the number of constituents that can be quantified and identified in samples varies from case to case [33,34,35]. 

Baloš et al. found polyfloral honey acidity values between 5.0 and 26.0 [36] and in the case of samples from Romania, values between 9.1 and 34.1 mqe/kg. The maximum value of moisture content is less than 20% (according to the Codex Alimentarius) and according to Baloš et al. [37] varies depending on the year between 15.6 and 19.6% for polyfloral honey. For the samples analyzed in the present study, the results range from 12.53 to 16.01%. The high water content of honey can lead to honey fermentation during storage [38].

The pH value of the honey samples was between 3.07 and 4.54, and together with the free acidity this prevents the growth of different microorganisms. The parameter values were overtaken only when the processing or storage was inadequate, also in the case of honey fermentation [39,40,41].

Water activity is an important factor, which provides information on food stability by preventing or limiting microbial growth. The osmotolerant yeasts are able to grow at a minimal water activity of 0.6 [38]. The water activity of honey in the investigated samples varies between 0.524 and 0.602. This range indicates an unsuitable environment for most bacteria. The results of the HMF content in the honey samples are mostly low. 

The values of electrical conductivity for Romanian honey range between 0.23 and 0.93 mS/cm, while Baloš et al. [36] found values of 0.09–0.74 mS/cm for Serbian honey. They also established a correlation between acidity and conductivity in the case of polyfloral honey.

**Figure 1:**
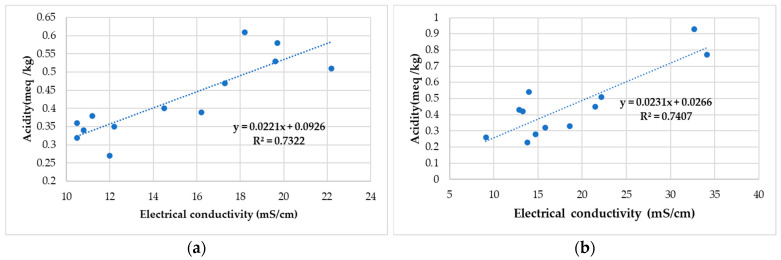
Correlation between acidity and electrical conductivity for polyfloral honey samples (**a**) from North-West and Central Romania and (**b**) from Alba County.

In Figure 1 the correlation between acidity and electrical conductivity for polyfloral honey samples collected for the study is presented.

Additionally, in the case of Romanian honey, an appreciable correlation was established between free acidity and electrical conductivity for polyfloral honey samples (R^2^ = 0.73 and R^2^= 0.74). High conductivity values indicate a high content of mineral substances.

According to STAS 784/3-2009 [42], the main physical and chemical characteristics and recommended limits for honey sold on the Romanian market are: humidity, max. 20%; acidity, max. 4 (mL NaOH sol. 1 N/100 g); reducing sugar is expressed as inverted sugar, min. 70%; easily hydrolyzable sugar is expressed as sucrose, max 5%; diastatic index, min. 6.5; ash, max. 0.5%; specific pollen grains, from the total number of pollen grains studied, min. 25%; hydroxymethylfurfural (HMF), max. 1.5 mg/100 g (honey packaged in a jar allows a HMF content of max. 4 mg per 100 g); colorimetric index, mm. (Pfund scale) max. 18; insoluble substances, max. 0.1, adulteration agents (artificial inverted sugar, industrial glucose or other substance from the hydrolysis of starch, gelatin, gum, cereals produced by flowers, dyes, synthetic sweeteners, etc.)—0. European legislation [29] comes with higher limits. In the case of heavy metals, the values are identical both at the European level (European Honey Directive of the European Honey Commission) and internationally (Codex Alimentarius Standard of F.A.O./O.M.S Commission), thus for Cu, the maximum is 0.50 mg/kg, Cd, the maximum is 0.02/(0.20) mg/kg, and Pb, the maximum is 0.20 mg/kg.

In general, high values of heavy metals in honey can come from industrial activities or as a result of intense car traffic. These activities pollute the atmospheric air. The metals reach the plants through the air and are finally found in honey. In our case, samples SS2 and SS4, which have high lead content, were collected from the Zlatna and Teiuș localities. Non-ferrous metals have been processed in Zlatna for a long time, and Teius is a junction Road railway station. Heavy metals (cadmium and lead) are used as bioindicators of honey contamination [43].

In their studies, Lianda et al., [44] discovered the fact that antioxidant activities and total phenolic contents are highly correlated.

**Figure 2:**
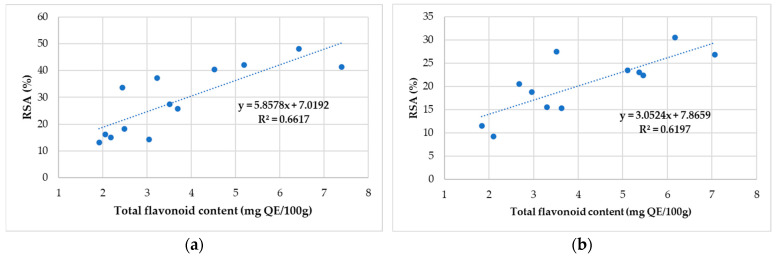
Correlation between antioxidant activity and total flavonoid content of the honey sample collected (**a**) from North-West and Central Romania and (**b**) from Alba County.

The correlation of RSA of 2,2-diphenyl-1-picrylhydrazyl with total flavonoid content for honey and propolis for all samples is presented in Figure 2a,b.

The lowest free radical-scavenging activity (RSA) in honey samples was observed in SS9, a sample collected from the Feneș, Alba County, while the highest was observed in SS13 from Baia de Arieș. It was found that sample SS10—Cugir had the lowest concentration of flavonoids compounds (1.84 mgQE/100 g), while the highest is registered in sample S4—Bistrița-Năsăud (7.39 mgQE/100 g). A moderate positive relationship between antioxidant activity and total flavonoids in the honey sample (R^2^ values between 0.62 and 0.66) was observed.

**Figure 3:**
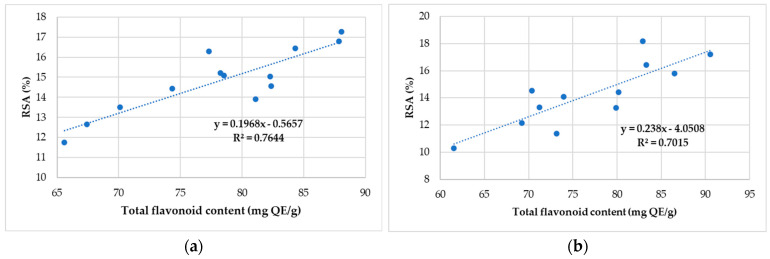
Correlation between antioxidant activity and total flavonoid content of the propolis sample collected (**a**) from North-West and Central Romania and (**b**) from Alba County.

Figure 3 illustrates the antioxidant activity vs. flavonoid content in the case of propolis samples taken in the study.

The antioxidant activity values vary between 10.29 (SS10—Cugir) and 18.19% (SS5—Șona), and for flavonoid content between 61.56 (SS10—Cugir) and 90.54 mQE/g (SS 4—Teiuș). Additionally, a significant and positive relationship occurs between the antioxidant activity and total flavonoid content for propolis samples from the North-West and Center of Romania, and Alba County.

Considering the values of the correlation coefficients, it is possible to suggest that flavonoid compounds are responsible for the antioxidant activity of the selected honey samples from Romania.

**Figure 4:**
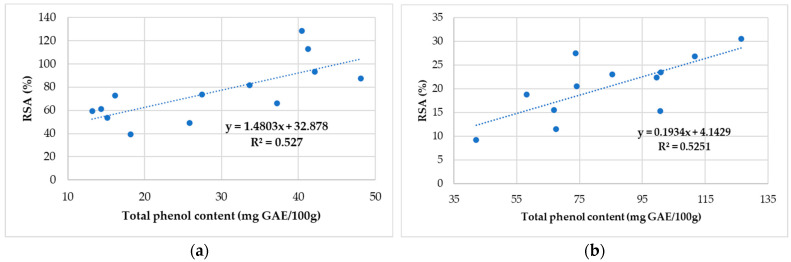
Correlation between antioxidant activity and total phenolic content of the honey sample collected (**a**) from North-West and Central Romania and (**b**) from Alba County.

**Figure 5:**
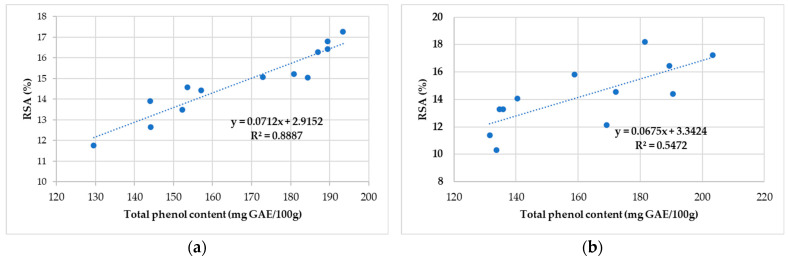
Correlation between antioxidant activity and total phenolic content for the propolis sample collected (**a**) from North-West and Central Romania and (**b**) from Alba County.

The correlation of total phenolic content with antioxidant activity for honey and propolis sample is presented in Figure 4a,b, and Figure 5a,b.

A weak correlation is observed between the RSA and the phenol content, where the values vary between 39.14 GAE/100 g (S6—Cluj) and 128.52 GAE/100 g (S5—Caraș-Severin). Correlation coefficients (R^2^ = 0.53) indicate variables (the antioxidant activity and total phenolic content honey sample) that can be considered moderately correlated.

Correlation coefficients between 0.55 and 0.89 indicate a fairly strong positive relationship regarding the RSA of 2,2-diphenyl-1-picrylhydrazyl in the examined propolis samples with the data obtained using the total phenolic compounds. 

We can conclude that the antioxidant activity of different samples of honey and propolis depends on their total phenolic and flavonoid concentration.

Other studies indicate a linear correlation between total phenolic and flavonoid content and the antioxidant activity of honey and honey products [25,45,46,47].

Regarding the antifungal effect of honey, it can be noticed that all the samples had an effect against the selected strains (Table 7 and Table 8). The diameters of the inhibition zones are different depending on the strain and on the honey source, varying between 7 and 12 mm. The most sensitive strains to the antifungal activity of honey were *Penicillium chrysogenum* and *Rhizopus stolonifer*. From the MIC analysis, it was observed that some honey samples can have an inhibitory effect even at dilutions of 1/32. Samples S4, S5 and S13 had the strongest antifungal effect. The honey from this North-West and Central region of Romania has a chemical composition and particular physicochemical properties described in our previous studies [48] and a strong antimicrobial effect confirmed by other studies in which the antibacterial effect of honey and propolis from this region was observed [27,49].

Our results confirm the fact that honey can have an antifungal effect on strains such as *Aspergillus* spp., *Candida* spp., *Penicillium* spp., *Rhizopus* spp. or *Fusarium* spp., as has been demonstrated by other previous studies on honey samples from some geographical areas such as Nigeria, Pakistan or India [17,50,51].

All aqueous extracts of propolis had an antifungal effect, with diameters varying between 15 and 28 mm (Table 9 and Table 10). The most sensitive strains to propolis activity were *P. chrysogenum* and *Fusarium oxisporumn*. The strongest antifungal effect was observed in samples S1, S4 and S5, with the last two being obtained from the same locations as honey samples S4 and S5, which also presented the strongest antifungal effect, confirming the fact that the geographical area and botanical origin can influence the antimicrobial properties of honey and propolis. Moreover, in the case of propolis, our study confirms previous studies on the effect of some propolis samples on fungal strains [28,52].

In the current study, we evaluated the antifungal effect of aqueous propolis extracts and not ethanolic ones, as they are usually used, in order to avoid the influence of ethanol on the diameters of the inhibition zones. However, the results regarding the antifungal effect of aqueous propolis extracts were positive even at a low concentration of 1.56 mg/mL in some samples.

The present paper highlighted the ability of honey and propolis samples to inhibit the growth of several species of fungi, which confirms their antifungal properties, making them potential candidates for application as antifungal agents.

## 4. Materials and Methods

### 4.1. Honey and Propolis Samples

**Figure 6:**
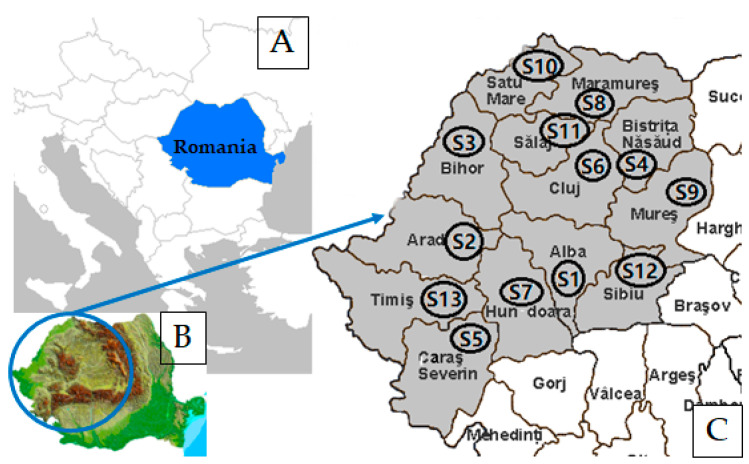
Romania’s position on the map of Europe (**A**); Geographical map of Romania, highlighting the sampling area (**B**); identification of the 13 counties for sampling (**C**).

Honey and propolis samples were collected from different locations in the North-West and Center of Romania in June–July 2021. Figure 6 shows the position of Romania on the map of Europe and the sampling points.

Table 14 lists the 13 samples of polyfloral honey and propolis, collected from beekeepers from different counties in the North-West and Center of Romania, and 12 samples from Alba County, that were used for analysis. The honey and propolis samples were collected directly from the producer and in each location, they were sampled from the same beekeeper. Each sample was collected in duplicate in a 200 g sterile container and kept in a dark place at 2–8 °C until tested. 

In the case of honey, initially, the samples were subjected to a low-level heat treatment (49.5 °C for 15 min) to reduce potential contamination and their microbial purity was checked. As a control, an artificial honey sample was used. It was prepared by dissolving 40.5 g of fructose, 33.5 g of glucose, 7.5 g of maltose and 1.5 g of sucrose in 17 mL of sterile deionized water, to emulate the proportions of the four predominant sugars in natural honey samples.

Aqueous extracts of propolis were used to determine the antifungal properties, according to the method described in our previous study [26]. Raw propolis (50 g) was mixed with 250 mL of water and the mixture was refluxed. After two series of centrifugations and filters, 80% of the initial mixture was evaporated, thus obtaining the aqueous extract of propolis, which was kept in a cool and dark place. A concentration of 0.1 g/mL was used for the analysis.

### 4.2. Physicochemical Analysis for Honey

The main physicochemical indicators that reflect the quality of honey and propolis were determined according to the methods proposed in “Harmonised methods of the international honey commission” [53], and the Romanian standard STAS 784/3-2009 [42].

#### 4.2.1. Water Content (Moisture) of Honey 

The water content (moisture) of honey was determined by refractive index (RI) at 20 °C using an ABBE refractometer. The water content (%) was set with the help of standard tables according to the refractive indices [42,54].

#### 4.2.2. pH Value and Free Acidity of Honey

An aqueous solution of 10 g sample in 75 mL water was prepared. The pH was measured with a WTW pH 340i pH meter. The solution was titrated with 0.1 M NaOH at pH 8.30 and the acidity was expressed in mEq/kg [53].

#### 4.2.3. Electrical Conductivity of Honey

The electrical conductivity of honey was measured at 20 °C in a solution of honey (20 g of dry matter in 100 mL of deionized water) using the conductometer Seven2Go (Mettler Toledo, Schwerzenbach, Switzerland) [53,54].

#### 4.2.4. The Water Activity (a_w_)

The water activity (a_w_) was determined with the Aquaspector apparatus AQS-2-TC (Nagy, Germany) [55].

#### 4.2.5. The Value in Pfund Scale for Color of Honey

The value in Pfund scale for color of honey was obtained using the Hanna Digital Colour Grader (Cluj-Napoca, Romania) [39].

#### 4.2.6. The Ash Content in Honey

The ash content in honey was measured by calcinating 10 g of honey to a constant mass and cooling it in a desiccator [8].

#### 4.2.7. Determination of HMF in Honey

For the determination of HMF in honey, 10 g of honey was dissolved in approximately 25 mL of distillate water and transferred to a 50 mL volumetric flask; 2 mL of honey solution and 5 mL of p-toluidine were placed in two test tubes. In one tube 1 mL of distilled water was added (reference solution) and in the other 1 mL of barbituric acid solution 0.5% (sample solution). The absorbance was read in 1 cm cuvettes at 550 nm with a Lambda 20 UV VIS Spectrophotometer (Perkin Elmer, Waltham, MA, USA). The HMF content was determined by the external standard method (p 99%, Sigma-Aldrich, Milan, Italy) and by using the proposed formula for the method [53].

#### 4.2.8. Determination of Cu, Pb and Cd

Voltammetric measurements were carried out with a Radiometer Pol 150 Polarographic Analyzer, connected to a MDE 150 polarographic stand, controlled with a PC via Trace Master 5 software. A hanging Mercury Drop Electrode (HMDE) was used as the working electrode (0.4 mm diameter, current ranges: 10 nA–10 μA), whereas a platinum rod and a Ag/AgCl electrode were used as auxiliary and reference electrodes, respectively. The supporting electrolyte used was 1 M HCl. The reagents used were: support electrolyte 1 M HCl, solution of Cu(II) 1000 mg/L, solution of Pb(II) 1000 mg/L, solution of Cd(II) 1000 mg/L, and a standard solution containing 50 mg/L Cu(II)-10 mg/L Pb(II)-1 mg/L Cd(II).

The honey samples were subjected to mineralization by microwave digestion. The amount subjected to mineralization was 1 g/sample, over which 6 mL of HNO_3_ (65%) was added. After mineralization, the samples were brought to a final volume of 10 mL with distilled water. The determination of the metal content consisted of the analysis of the sample followed by three additions of 50 μL each of the standard solution.

#### 4.2.9. Determination of Total Phenolic Content (TPC)

For the determination of Total Phenolic Content (TPC), the Folin–Ciocalteu method was used [56,57]. Total phenol content was determined by interpolating the absorbance of the honey based on a calibration curve constructed with standard Gallic acid, with a purity of 98%.

#### 4.2.10. Total Flavonoid Content (TFC) 

For the determination of Total flavonoid content, 2.5 g of honey and 0.5 mL of AlCl_3_ 5% were dissolved in distilled water in a 25 mL volumetric flask. After 30 min in a dark place, the absorbance readings at 425 nm were determined. The total flavonoid content was established using a standard curve, with quercetin (producer (0–50 mg/L) as the standard. The mean of three readings was used and expressed as mg of quercetin equivalents (QE)/100 g of honey [58].

#### 4.2.11. DPPH Radical Scavenging Activity 

The DPPH scavenging activity was measured as described by Meda et al. [59]. Initially, the DPPH reagent was prepared with a concentration of 0.02 mg/mL in methanol. 

Honey samples were dissolved in methanol at a concentration ranging from 5 to 60 mg/mL. 

Honey was diluted in methanol and filtered with a 0.45 µm sterile Millex syringe filter (producer Sigma-Aldrich); 0.8 mL of a 2.5% honey solution (*w/v*) was added to 2.7 mL of 0.024 mg/mL 2,2-diphenyl-1-picrylhydrazyl in methanol and homogenized and kept in a dark place for 30 min. The absorbance was read at 517 nm using a Lambda 20 UV VIS Spectrophotometer (Perkin Elmer, Waltham, MA, USA). The radical scavenging activity (RSA) was calculated as the percentage of DPPH discoloration using the equation RSA (%) = [(A_DPPH_ − A_S_)/A_DPPH_] × 100. (A_DPPH_ − absorbance of DPPH solutions without honey; A_S:_ absorbance of DPPH solutions without honey).

### 4.3. Physicochemical Analysis for Propolis

#### 4.3.1. Moisture

The moisture content of the samples was determined using an A&D ML50 Moisture Analyzer (San Jose, CA, USA) [60].

#### 4.3.2. Ash (Total Mineral Substances)

In a crucible, 5 g of propolis is completely carbonized on a Bunsen burner, then calcined at 550 ± 25 °C for 10–12 h until constant mass [8]. 

#### 4.3.3. Wax (Extractable Substances)

The determination was made from dry propolis (5 g) after repeated extraction with petroleum ether at 40–60 °C for 3 h in a Soxhlet extractor. After the waxes were separated and the solvent removed, the samples were placed in an oven at 100 °C for 3 h and cooled until they reached a constant weight [8].

#### 4.3.4. Qualitative Identification of Flavones’ Presence

Dried raw propolis (5 g) was ground to a fine powder in a mixer and dissolved in 20 mL of 96% ethanol. After 3 h of constant stirring, the extract was filtered and heated for accelerated evaporation of the alcohol. Borax (5 g) and distilled water (10 mL) were added bit by bit and the mixture was homogenized. The milky liquid was filtered and a few drops were added to filter paper. The presence of flavones was confirmed by color changes detected on the yellow spots; reddish brown when adding uranyl nitrate crystals, and gray when adding crystals of ferric sulfate [8].

#### 4.3.5. Identification of Aromatic Acids

The solution prepared in order to identify the flavones (5 mL) was precipitated with sulfuric acid (1:10). Peroxide-free ethyl ether (10 mL) was added and the mixture was homogenized for one minute and left to allow separation of the ether/aqueous layers. The extraction ether (upper layer) was collected. Ethyl ether (10 mL) was added to the lower layer and the previous operation was repeated; the extraction ether was also collected. The ether solution was filtered on anhydrous sodium sulfate and then evaporated to dryness. The addition of 2 N NaOH and KMnO_4_ drops under moderate heating produced the aromas of bitter almonds (benzoic aldehyde) and cinnamon (cinnamic aldehyde) [8].

#### 4.3.6. Quantification of the Phenolic Compounds

For this determination, the Folin–Ciocalteu method was used [61,62]. Dried raw propolis was grounded in a mixer to a fine powder, dissolved and homogenized in ethanol and filtered. An equivalent quantity of Folin–Ciocalteu reagent was added. The absorbance was measured against a blank (distilled water) at 765 nm with a Lambda 20 UV VIS Spectrophotometer (Perkin Elmer UV/VIS, Washington D.C., USA). The total phenolic concentrations were compared to a standard curve of gallic acid.

#### 4.3.7. Determination of Flavonoid Content

The flavonoid content was conducted by the Aluminum Chloride Colorimetric method [61,63,64]. Ethanoic propolis extracts and quercetin ethanoic dilutions were used as the standard to produce the calibration curve. The diluted standard quercetin solutions and the extracted solutions were separately mixed with 95% ethanol and aluminum chloride and then incubated at room temperature. The absorbance was measured at 415 nm against a blank with a Lambda 20 UV VIS Spectrophotometer (Perkin Elmer UV/VIS, Washington, DC, USA). The concentration of the total flavonoid content was derived from the calibration plot. The mean of three readings was used and the total flavonoid content was expressed as quercetin equivalents (mg EQ/g).

#### 4.3.8. The Antioxidant Activity of Propolis

The raw propolis samples were macerated and continuously homogenized for 24 h in 70% ethanol solution (1:100 *w/v*), and then evaporated to dryness. A reaction mixture containing 2,2-diphenyl-1-picrylhydrazyl (DPPH) 0.1 mM ethanoic solution and 0.6 mg/mL propolis solution was prepared. The absorbance was measured in a quartz cuvette (1 cm^3^) at λ = 515 nm with a Lambda 20 UV VIS Spectrophotometer (Perkin Elmer UV/VIS, Washington D.C., USA). Absorbance (A) was measured at the initiation of the reaction, then after 10 and 20 min. The antioxidant activity was calculated using the formula: %RSA = (A_DPPH_ − A_sample_)/A_DPPH_ ×100 [65,66].

### 4.4. Antifungal Activity

#### 4.4.1. Micro-Organisms and Culture Conditions

To test the antifungal properties of both the bee honey and propolis aqueous extracts, a disk diffusion method was used according to CLSI-recommended procedures [67]. Antifungal activity tests were performed against six reference strains, some of which are pathogenic for humans, and plants, or can be indicators of microbiological contamination of food: *Aspergillus brasiliensis (niger)* (ATCC 16404), *Aspergillus flavus* (ATCC 9643), *Candida albicans* (ATCC 10231), *Penicillium chrysogenum* (ATCC 10106), *Rhizopus stolonifer* (ATCC 14037) and *Fusarium oxysporum* (ATCC 48112) were provided by Thermo Fisher Scientific Inc. (Waltham, MA, USA) and MicroBioLogics Inc. (St. Cloud, MN, USA). Direct colony suspensions of overnight cultures were diluted in sterile normal saline and turbidity was adjusted to 0.5 McFarland. Sabouraud 4% dextrose agar (Merck KgaA, Darmstadt, Germany) was used as a culture medium in Petri dishes with a depth of ~4 mm (25 mL/plate). The surface of the Petri dishes was inoculated by flooding with 1 mL culture, then spread on the surface. After inoculation, the plates were kept for 15 min at 37 ^◦^C, to absorb the inoculum in the agar.

#### 4.4.2. Determination of the Antifungal Properties—Agar Disk Diffusion Method

Before use, the honey samples were heated to 40–45 °C for the complete dissolution of the crystals. Using a sterile stainless-steel tube, circles with a diameter of 6.0 mm were made in the medium of the Petri dishes, then each hole was filled with a sample of honey (including the prepared artificial honey), the inoculated amounts being 150 μL. The plates were incubated for 5 days at 25 ± 1 °C, with the lids up.

From each propolis aqueous extract of 0.1 g/mL concentration, 50 μL was added onto sterile ~6 mm filter paper discs. The disks were placed sterile on the surface of the culture medium, kept for 120 min at 5 °C, then incubated for 5 days at 25 ± 1 °C for fungal growth.

Antifungal activity was determined by measuring the inhibition zones (in mm) produced by each sample of honey or propolis with a DIN 862 ABS digital caliper (Fuzhou Conic Industrial Co. Ltd., Fuzhou, China) with ±0.01 mm precision. 

#### 4.4.3. Minimum Inhibitory Concentration (MIC) of the Honey and Propolis Samples

For the honey samples, MIC values were determined using an initial dilution method with 10 g graduated doses (*w/v*) of different kinds of honey dissolved in sterile deionized water to obtain dilutions of 1/1, 1/4, 1/16, 1/32 and 1/64. The aqueous propolis extracts were mixed with deionized water (*v/v*) to obtain the final dilutions of 1/1, 1/4, 1/8, 1/16, 1/32, 1/64 and 1/128. The antifungal activity of the diluted samples of honey and propolis extracts was evaluated by the disc diffusion method as described above (see Section 4.4.2). The MIC was considered to be the lowest concentration at which microbial growth was inhibited.

### 4.5. Statistical Analysis

Two-way analysis of variance (ANOVA) tests was used to assess differences and interactions between the diameter of inhibition zones, for different strains of honey and propolis, and their geographic origin [68].

## 5. Conclusions

The study allowed the analysis of honey and propolis samples collected from beekeepers in the North-West and Center of Romania, to determine the chemical and physical characteristics, as well as the content of heavy metals: lead, copper and cadmium, as indicators of the quality of the samples and environmental pollution. The investigated parameters are within the limits imposed by the legislation, with the exception of lead in the Zlatna and Teiuş localities, considered industrialized areas or areas with intense traffic.

The content of phenols and flavonoids In honey samples fluctuates depending on the sampling area, but there are no major differences between the samples collected from Alba County, compared to the Center and North-West of the country. In the case of propolis, the North-West and Central Romanian samples from the counties exhibited the highest content of bioactive compounds compared to those from Alba county.

All honey and propolis samples had an antifungal effect, but differed depending on the area of origin and the type of strain, with the most sensitive strains being *Penicillium chrysogenum, Rhizopus stolonifer* and *Fusarium oxysporum*.

There was a significant correlation between the acidity and electrical conductivity of polyfloral honey and the antioxidant activity with total phenolic and flavonoid content. Statistical analysis (the two-way ANOVA analysis for antifungal activity) shows the connection between the geographical origin of the honey and propolis samples, the microbial strains used and the antifungal activity. Variance analysis shows that both strain type and geographic origin of honey and propolis influence the diameter of inhibition.

## Figures and Tables

**Table 1:** Physicochemical parameters of honey samples from North-West and Central Romania.

Sample No.	Moisture Content (%)	pH	Acidity (meq/kg)	Electrical Conductivity (mS/cm)	a_w_	Pfund (mm)
S1	15.78 ± 0.9	4.25 ± 0.5	22.2 ± 4.7	0.51 ± 0.02	0.548 ± 0.035	52
S2	13.05 ± 0.6	3.88 ± 0.3	11.2 ± 4.6	0.38 ± 0.02	0.536 ± 0.022	52
S3	14.67 ± 0.7	3.81 ± 0.3	12.0 ± 3.2	0.27 ± 0.00	0.571 ± 0.028	57
S4	15.04 ± 0.9	3.93 ± 0.2	10.8 ± 2.4	0.34 ± 0.01	0.588 ± 0.034	51
S5	14.66 ± 0.6	3.27 ± 0.1	10.5 ± 3.0	0.36 ± 0.02	0.571 ± 0.022	53
S6	13.48 ± 0.8	4.08 ± 0.3	14.5 ± 4.2	0.40 ± 0.01	0.532 ± 0.010	56
S7	14.54 ± 0.7	4.02 ± 0.1	17.3 ± 3.7	0.47 ± 0.02	0.549 ± 0.021	57
S8	14.82 ± 0.8	3.74 ± 0.3	19.7 ± 4.4	0.58 ± 0.02	0.545 ± 0.025	49
S9	13.27 ± 0.5	3.49 ± 0.3	12.2 ± 4.9	0.35 ± 0.01	0.524 ± 0.029	55
S10	14.45 ± 0.8	3.55 ± 0.2	18.2 ± 4.5	0.61 ± 0.03	0.562 ± 0.028	53
S11	14.56 ± 0.9	3.18 ± 0.3	16.2 ± 3.8	0.39 ± 0.00	0.555 ± 0.019	50
S12	15.63 ± 0.8	3.92 ± 0.3	10.5 ± 3.2	0.32 ± 0.02	0.579 ± 0.023	54
S13	14.29 ± 0.9	3.83 ± 0.2	19.6 ± 3.1	0.53 ± 0.01	0.568 ± 0.024	49

**Table 2:** Physicochemical parameters of honey samples from Alba County.

Sample No.	Moisture Content (%)	pH	Acidity (meq/kg)	Electrical Conductivity (mS/cm)	a_w_	Pfund (mm)
SS1	15.78 ± 0.9	4.25 ± 0.5	22.2 ± 4.7	0.51 ± 0.02	0.548 ± 0.035	52
SS2	12.64 ± 0.6	4.06 ± 0.2	13.8 ± 3.2	0.23 ± 0.03	0.552 ± 0.024	50
SS3	15.33 ± 1.0	3.62 ± 0.3	9.1 ± 4.0	0.26 ± 0.00	0.602 ± 0.038	58
SS4	13.75 ± 0.1	3.53 ± 0.1	14.7 ± 3.1	0.28 ± 0.00	0.567 ± 0.041	54
SS5	13.64 ± 0.8	4.54 ± 0.4	12.9 ± 2.6	0.43 ± 0.02	0.551 ± 0.023	49
SS6	13.07 ± 0.7	3.35 ± 0.3	13.3 ± 6.5	0.42 ± 0.01	0.584 ± 0.019	56
SS7	16.01 ± 1.2	3.07 ± 0.2	21.5 ± 5.8	0.45 ± 0.02	0.536 ± 0.015	59
SS8	13.75 ± 0.7	3.94 ± 0.2	18.6 ± 3.4	0.33 ± 0.01	0.575 ± 0.026	48
SS9	12.53 ± 0.3	3.71 ± 0.1	15.8 ± 2.7	0.32 ± 0.00	0.539 ± 0.033	56
SS10	14.96 ± 0.6	3.82 ± 0.3	14.0 ± 9.1	0.54 ± 0.01	0.593 ± 0.025	51
SS11	13.47 ± 0.8	3.79 ± 0.2	32.7 ± 4.2	0.93 ± 0.01	0.547 ± 0.017	57
SS12	16.05 ± 1.4	4.24 ± 0.4	34.1 ± 3.5	0.77 ± 0.02	0.536 ± 0.012	55

**Table 3:** The chemical parameters of the honey samples from North-West and Central of Romania.

Sample No.	Ash g/100 g	HMF (mg/kg)	Phenols (mg GAE/100 g)	Flavonoids (mg QE/100 g)	Lead, mg/kg	Copper, mg/kg	Cadmium, mg/kg	RSA (%)
S1	0.19	0.9 ± 0.2	73.80 ± 0.25	3.51 ± 0.52	ND	ND	ND	27.45
S2	0.28	1.2 ± 0.4	53.67 ± 0.49	2.18 ± 0.17	ND	0.204 ± 0.006	ND	15.12
S3	0.31	1.1 ± 0.3	61.38 ± 0.52	3.04 ± 0.26	ND	ND	ND	14.32
S4	0.27	3.1 ± 0.2	113.03 ± 0.90	7.39 ± 0.11	0.042 ± 0.004	ND	ND	41.27
S5	0.19	0.1 ± 0.3	128.52 ± 0.98	4.52 ± 0.23	ND	0.118 ± 0.003	0.006 ± 0.002	40.43
S6	0.21	4.7 ± 0.2	39.41 ± 0.29	2.49 ± 0.09	0.120 ± 0.005	ND	ND	18.16
S7	0.25	2.5 ± 0.4	93.09 ± 1.12	5.19 ± 0.15	ND	0.107 ± 0.004	0.002 ± 0.001	42.13
S8	0.22	1.7 ± 0.2	81.60 ± 0.84	2.44 ± 0.13	ND	ND	ND	33.65
S9	0.19	0.4 ± 0.2	59.45 ± 0.87	1.92 ± 0.04	0.051 ± 0.003	ND	ND	13.16
S10	0.18	1.2 ± 0.3	72.74 ± 0.51	2.06 ± 0.51	ND	0.214 ± 0.005	ND	16.13
S11	0.24	2.3 ± 0.3	66.07 ± 0.44	3.23 ± 0.08	0.106 ± 0.008	ND	ND	37.2
S12	0.31	5.4 ± 0.4	49.24 ± 0.63	3.69 ± 0.03	ND	ND	0.004 ± 0.001	25.84
S13	0.23	0.2 ± 0.1	87.49 ± 1.21	6.43 ± 0.16	ND	0.169 ± 0.007	ND	48.09

RSA—radical-scavenging activity; ND—not detectable (<0.05 mg/kg Cu; 0.001 mg/kg Cd; 0.01 mg/kg Pb).

**Table 4:** The chemical parameters of the honey samples from Alba County.

Sample No.	Ash g/100 g	HMF (mg/kg)	Phenols (mg GAE/100 g)	Flavonoids (mg QE/100 g)	Lead, mg/kg	Copper, mg/kg	Cadmium, mg/kg	RSA (%)
SS1	0.19	0.9 ± 0.2	73.80 ± 0.25	3.51 ± 0.52	ND	ND	ND	27.45
SS2	0.34	4.0 ± 0.3	100.72 ± 1.20	3.63 ± 0.20	0.334 ± 0.021	ND	ND	15.32
SS3	0.19	2.8 ± 0.4	126.53 ± 1.10	6.17 ± 0.17	0.176 ± 0.013	ND	ND	30.48
SS4	0.22	0.4 ± 0.1	111.68 ± 0.92	7.06 ± 0.03	0.471 ± 0.043	ND	ND	26.79
SS5	0.17	0.2 ± 0.1	100.81 ± 0.54	5.11 ± 0.05	ND	0.125 ± 0.015	ND	23.46
SS6	0.18	0.9 ± 0.2	99.55 ± 0.41	5.46 ± 0.15	ND	ND	0.002 ± 0.001	22.39
SS7	0.11	2.3 ± 0.1	85.47 ± 0.59	5.37 ± 0.29	ND	0.138 ± 0.011	0.003 ± 0.001	23.04
SS8	0.20	1.6 ± 0.3	66.84 ± 0.22	3.30 ± 0.18	ND	0.100 ± 0.008	0.007 ± 0.001	15.5
SS9	0.18	0.5 ± 0.2	42.10 ± 0.11	2.10 ± 0.03	0.062 ± 0.03	ND	ND	9.27
SS10	0.17	3.1 ± 0.2	67.52 ± 0.63	1.84 ± 0.11	ND	0.496 ± 0.081	0.011 ± 0.002	11.46
SS11	0.21	0.8 ± 0.3	74.06 ± 0.48	2.68 ± 0.04	ND	0.411 ± 0.042	ND	20.55
SS12	0.32	0.7 ± 0.3	58.12 ± 0.87	2.96 ± 0.80	ND	ND	0.005 ± 0.001	18.83

RSA—radical-scavenging activity; ND—not detectable (<0.05 mg/kg Cu; 0.001 mg/kg Cd; 0.01 mg/kg Pb).

**Table 5:** Characterization of propolis samples from North-West and Central of Romania.

Sample No.	Moisture (%)	Ash (g/100 g)	Wax (%)	Phenols (mg GAE/g)	Flavonoids (mg QE/g)	RSA (%)
S1	8.04 ± 0.12	3.14 ± 0.07	25.84 ± 0.57	189.4 ± 5.82	84.31 ± 0.09	16.44
S2	6.34 ± 0.63	2.85 ± 0.08	37.18 ± 0.81	180.8 ± 4.54	78.26 ± 0.07	15.21
S3	7.64 ± 0.27	2.96 ± 0.04	40.56 ± 1.06	172.9 ± 3.25	78.55 ± 0.08	15.08
S4	9.11 ± 0.89	3.15 ± 0.06	33.22 ± 0.38	189.5 ± 4.83	87.84 ± 0.11	16.79
S5	7.56 ± 0.28	3.28 ± 0.09	46.33 ± 1.05	193.4 ± 7.22	88.06 ± 0.08	17.27
S6	4.81 ± 0.80	2.55 ± 0.05	37.41 ± 0.58	129.6 ± 3.58	65.59 ± 0.09	11.75
S7	6.52 ± 0.46	2.73 ± 0.03	31.19 ± 0.71	184.3 ± 6.04	82.27 ± 0.25	15.04
S8	7.32 ± 0.54	2.69 ± 0.04	32.52 ± 0.44	152.2 ± 6.80	70.10 ± 0.16	13.50
S9	5.43 ± 0.82	3.08 ± 0.03	28.92 ± 0.67	157.1 ± 5.57	74.35 ± 0.36	14.43
S10	7.05 ± 0.37	2.62 ± 0.02	34.24 ± 0.96	186.9 ± 6.88	77.33 ± 0.21	16.28
S11	6.27 ± 0.91	3.10 ± 0.05	39.47 ± 1.05	144.2 ± 5.51	67.41 ± 0.14	12.66
S12	7.63 ± 0.57	2.42 ± 0.08	27.65 ± 0.73	153.5 ± 4.78	82.38 ± 0.27	14.57
S13	8.18 ± 0.62	2.67 ± 0.07	38.15 ± 0.92	144.0 ± 2.09	81.09 ± 0.98	13.92

GAE—gallic acid equivalents; QE—quercetin equivalents; RSA—radical-scavenging activity.

**Table 6:** Characterization of propolis samples from Alba County.

Sample No.	Moisture (%)	Ash (g/100 g)	Wax (%)	Phenols (mg GAE/g)	Flavonoids (mg QE/g)	RSA (%)
SS1	8.04 ± 0.12	3.14 ± 0.07	25.84 ± 0.57	189.4 ± 5.82	84.31 ± 0.09	16.44
SS2	7.46 ± 0.06	3.05 ± 0.07	34.07 ± 1.12	172.2 ± 6.14	70.37 ± 0.03	14.54
SS3	7.38 ± 0.39	2.88 ± 0.08	32.71 ± 0.71	158.8 ± 5.27	86.48 ± 0.12	15.81
SS4	8.21 ± 0.64	3.22 ± 0.14	30.15 ± 0.57	203.3 ± 7.28	90.54 ± 0.06	17.22
SS5	6.78 ± 0.17	3.12 ± 0.05	28.92 ± 0.68	181.5 ± 6.10	82.92 ± 0.07	18.19
SS6	8.06 ± 0.60	2.24 ± 0.06	31.64 ± 0.27	134.7 ± 4.09	71.24 ± 0.02	13.30
SS7	7.86 ± 0.68	3.20 ± 0.09	29.38 ± 0.63	190.6 ± 5.26	80.19 ± 0.01	14.41
SS8	7.15 ± 0.82	2.49 ± 0.03	30.79 ± 1.01	169.1 ± 8.39	69.23 ± 0.04	12.15
SS9	6.70 ± 0.93	2.77 ± 0.02	27.67 ± 0.64	135.9 ± 7.42	79.89 ± 0.44	13.27
SS10	7.65 ± 0.37	2.72 ± 0.04	31.05 ± 0.91	133.7 ± 6.97	61.56 ± 0.59	10.29
SS11	6.99 ± 0.70	3.32 ± 0.08	31.26 ± 0.67	131.5 ± 3.01	73.15 ± 0.41	11.38
SS12	7.72 ± 0.44	2.89 ± 0.07	33.45 ± 0.85	140.4 ± 5.31	73.97 ± 0.29	14.07

GAE—gallic acid equivalents; QE—quercetin equivalents; RSA—radical-scavenging activity.

**Table 7:** Diameters of the inhibition zones on the fungal strains for honey samples from North-West and Central Romania.

No.	Strain	Sample Inhibition Diameter Area (mm)												
		S1	S2	S3	S4	S5	S6	S7	S8	S9	S10	S11	S12	S13
1	*A. niger*	9	9	9	10	10	9	8	9	8	9	8	9	10
2	*A. flavus*	9	9	9	10	9	9	8	9	9	9	9	9	10
3	*C. albicans*	8	9	8	9	10	8	8	8	7	8	7	8	9
4	*P. chrysogenum*	10	12	11	12	11	10	9	10	9	11	9	11	12
5	*R. stolonifer*	9	12	11	12	10	8	9	9	9	12	9	11	11
6	*F. oxysporum*	8	10	9	11	11	9	8	10	7	10	8	9	11

**Table 8:** Diameters of the inhibition zones on the fungal strains for honey samples from Alba County.

No.	Strain	Sample Inhibition Diameter Area (mm)											
		SS1	SS2	SS3	SS4	SS5	SS6	SS7	SS8	SS9	SS10	SS11	SS12
1	*A. niger*	9	8	9	9	10	8	9	10	9	11	9	10
2	*A. flavus*	9	10	9	9	11	9	9	8	10	10	9	9
3	*C. albicans*	8	9	8	8	9	9	10	9	8	8	9	10
4	*P. chrysogenum*	10	11	10	12	10	12	9	11	8	12	10	9
5	*R. stolonifer*	9	11	12	10	10	9	8	10	9	11	9	10
6	*F. oxysporum*	8	9	10	9	11	10	9	9	8	9	7	8

**Table 9:** Diameters of the inhibition zones in fungal strains for propolis aqueous extract samples from North-West and Central of Romania.

No.	Strain	Sample Inhibition Diameter Area (mm)												
		S1	S2	S3	S4	S5	S6	S7	S8	S9	S10	S11	S12	S13
1	*A. niger*	24	21	19	23	26	17	18	16	22	15	19	18	16
2	*A. flavus*	26	20	17	25	27	15	18	15	20	16	17	20	18
3	*C. albicans*	22	19	18	22	22	19	21	20	19	21	18	20	19
4	*P. chrysogenum*	27	24	22	26	27	23	24	25	23	25	22	26	24
5	*R. stolonifer*	24	22	20	26	25	21	23	22	22	24	20	23	21
6	*F. oxysporum*	28	23	24	25	26	23	22	27	21	25	23	26	22

**Table 10:** Diameters of the inhibition zones on the fungal strains for propolis aqueous extract samples from Alba County.

No.	Strain	Sample Inhibition Diameter Area (mm)											
		SS1	SS2	SS3	SS4	SS5	SS6	SS7	SS8	SS9	SS10	SS11	SS12
1	*A. niger*	24	20	18	24	26	19	25	17	21	16	19	20
2	*A. flavus*	26	22	16	25	27	17	20	15	21	18	18	19
3	*C. albicans*	22	18	20	21	23	17	22	21	18	20	16	20
4	*P. chrysogenum*	27	23	22	25	27	21	27	24	25	26	20	23
5	*R. stolonifer*	24	22	21	26	25	19	25	23	20	22	21	19
6	*F. oxysporum*	28	25	25	27	25	21	24	26	22	25	24	24

**Table 11:** Statistical bifactorial analysis of variance for samples from North-West and Central Romania.

Dispersion Sum of the Diameters of Inhibition Zones		Quadratic Sum	Degrees of Freedom (ν)	Variance (s^2^)	F_computed_	F_0.05_
Between honey samples	(S_2_–S_4_)	52.12	*m* − 1 = 5	s^2^_1,H_ = 10.42	10.42	2.37
Between propolis extracts		299.79		s^2^_1,P_ = 18.40	18.40	
Between strains, H	(S_3_–S_4_)	45.53	*n* − 1 = 12	s^2^_2,H_ = 3.79	9.18	1.92
Between strains, P		350.56		s^2^_2,P_ = 8.96	8.96	
Residual, H	Sr	24.79	(*m* − 1)(*n* − 1) = 60	s^2^_r,H_ = 0.41	-	-
Residual, P		195.43		s^2^_r,P_ = 3.25	-	-

**Table 12:** Statistical bifactorial analysis of variance for samples from Alba County.

Dispersion Sum of the Diameters of Inhibition Zones		Quadratic Sum	Degrees of Freedom (ν)	Variance (s^2^)	F_computed_	F_0.05_
Between honey samples	(S_2_–S_4_)	16.15	*m* − 1 = 5	s^2^_1,H_ = 3.23	3.53	2.38
Between propolis extracts		323		s^2^_1,P_ = 64.60	19.08	
Between strains, H	(S_3_-S_4_)	20.90	*n* − 1 = 11	s^2^_2,H_ = 1.90	2.07	1.97
Between strains, P		250.83		s^2^_2,P_ = 22.80	6.73	
Residual, H	Sr	50.26	(*m* − 1)(*n* − 1) = 55	s^2^_r,H_ = 0.91	-	-
Residual, P		186.16		s^2^_r,P_ = 3.38	-	-

**Table 13:** Pearson’s correlation coefficients between the diameter of the inhibition zone and the flavonoid/phenol content for the studied microbial strains.

Microbial Strains	Flavonoids				Phenols			
	Honey		Propolis		Honey		Propolis	
	N-V/Center	Alba	N-V/Center	Alba	N-V/Center	Alba	N-V/Center	Alba
*A. niger*	0.545	−0.395	0.549	0.699	0.513	−0.392	0.551	0.711
*A. flavus*	0.481	−0.132	0.789	0.541	0.213	0.045	0.644	0.618
*C. albicans*	0.509	−0.004	0.696	0.415	0.630	−0.129	0.710	0.732
*P. chrysogenum*	0.388	0.279	0.697	0.250	0.216	0.461	0.559	0.592
*R. stolonifer*	0.266	0.073	0.728	0.465	0.172	0.42	0.726	0.888
*F. oxisforumn*	0.489	0.599	0.313	0.317	0.531	0.650	0.362	0.694

**Table 14:** The origin of the honey and propolis samples.

Sample	County of Origin	Areal	Sub-Sample	Area
S1	Alba	Mountainous	Alba County	
S2	Arad	Plain	SS1	Blaj
S3	Bihor	Hilly	SS2	Zlatna
S4	Bistrița-Năsăud	Mountainous	SS3	Alba Iulia
S5	Caraș-Severin	Hilly	SS4	Teius
S6	Cluj	Hilly	SS5	Șona
S7	Hunedoara	Sub-mountainous	SS6	Berghin
S8	Maramureș	Mountainous	SS7	Crăciunelu de Jos
S9	Mureș	Hilly	SS8	Sântimbru
S10	Satu Mare	Hilly	SS9	Feneș
S11	Sălaj	Sub-mountainous	SS10	Cugir
S12	Sibiu	Sub-mountainous	SS11	Abrud
S13	Timiș	Plain	SS12	Baia de Aries
